# ATM, ATR and DNA-PKcs kinases—the lessons from the mouse models: inhibition ≠ deletion

**DOI:** 10.1186/s13578-020-0376-x

**Published:** 2020-01-29

**Authors:** Demis Menolfi, Shan Zha

**Affiliations:** 1 Institute for Cancer Genetics, College of Physicians & Surgeons, Columbia University, New York, NY 10032 USA; 2 Department of Pathology and Cell Biology, College of Physicians & Surgeons, Columbia University, New York, NY 10032 USA; 3 Division of Pediatric Oncology, Hematology and Stem Cell Transplantation, Department of Pediatrics, College of Physicians & Surgeons, Columbia University, New York, NY 10032 USA

**Keywords:** DNA damage response, ATM, DNA-PKcs, ATR, Kinase inhibition, Double-strand breaks (DSBs), Single-strand DNA (ssDNA), Lymphocyte development

## Abstract

DNA damage, especially DNA double strand breaks (DSBs) and replication stress, activates a complex post-translational network termed DNA damage response (DDR). Our review focuses on three PI3-kinase related protein kinases—ATM, ATR and DNA-PKcs, which situate at the apex of the mammalian DDR. They are recruited to and activated at the DNA damage sites by their respective sensor protein complexes—MRE11/RAD50/NBS1 for ATM, RPA/ATRIP for ATR and KU70–KU80/86 (XRCC6/XRCC5) for DNA-PKcs. Upon activation, ATM, ATR and DNA-PKcs phosphorylate a large number of partially overlapping substrates to promote efficient and accurate DNA repair and to coordinate DNA repair with other DNA metabolic events (*e.g.*, transcription, replication and mitosis). At the organism level, robust DDR is critical for normal development, aging, stem cell maintenance and regeneration, and physiological genomic rearrangements in lymphocytes and germ cells. In addition to endogenous damage, oncogene-induced replication stresses and genotoxic chemotherapies also activate DDR. On one hand, DDR factors suppress genomic instability to prevent malignant transformation. On the other hand, targeting DDR enhances the therapeutic effects of anti-cancer chemotherapy, which led to the development of specific kinase inhibitors for ATM, ATR and DNA-PKcs. Using mouse models expressing kinase dead ATM, ATR and DNA-PKcs, an unexpected structural function of these kinases was revealed, where the expression of catalytically inactive kinases causes more genomic instability than the loss of the proteins themselves. The spectrum of genomic instabilities and physiological consequences are unique for each kinase and depends on their activating complexes, suggesting a model in which the catalysis is coupled with DNA/chromatin release and catalytic inhibition leads to the persistence of the kinases at the DNA lesion, which in turn affects repair pathway choice and outcomes. Here we discuss the experimental evidences supporting this mode of action and their implications in the design and use of specific kinase inhibitors for ATM, ATR and DNA-PKcs for cancer therapy.

## Background

The genomic DNA in mammalian cells is constantly challenged by base modifications and strand breaks [1]. Among them, DNA double-strand breaks (DSBs) and extended single stranded DNA (ssDNA) activate a network of post-translational modifications, including, but not limited to, phosphorylation, which are broadly termed DNA damage responses (DDR). Notably, base modifications, base crosslinks or DNA mismatches are detected and repaired by base excision repair (BER), nucleotide excision repair (NER) and mismatch repair (MMR), respectively [2–4]. Although ssDNA nicks and short patches of ssDNA might be generated during the repair, they generally do not elicit the type of DDR discussed here, unless the damage is converted to DSBs or extended ssDNA upon transcription or replication. This selective response to DNA double strand breaks and extended ssDNA allows the cells to tolerate physiological DNA nicks or single strand breaks during normal transcription and replication. For this review, we focus on three phosphoinositide 3 kinase-related protein kinases (PI3KKs)—ataxia-telangiectasia mutated (ATM), ATM and RAD3-related (ATR) and DNA-dependent protein kinase catalytic subunit (DNA-PKcs) [5], which reside at the apex of the mammalian DDR and phosphorylate an overlapping spectrum of substrates. In particular, we focus on the early events upon the kinase activation, namely the activation associated structural function of ATM, ATR and DNA-PKcs, by comparing the phenotypes of the mouse models expressing kinase-dead (KD) mutants of ATM, ATR and DNA-PKcs vs their respective null mutation. In terms of the DNA lesions, DNA-PKcs is activated by DSBs ends [6], ATR by extended ssDNA [7] and ATM by potentially diverse DNA structures including chromosomal DNA DSBs and others [8].

In mammals, DSBs can be generated during physiological processes, like V(D)J recombination in developing lymphocytes and meiosis recombination in germ cells, or as byproducts of genotoxic challenges (e.g., replication blockers, ionizing radiation (IR)) [9]. Extended ssDNA can also be generated during DNA metabolism that requires the separation of DNA-double helices, such as replication and transcription. The activation of ATM, ATR and DNA-PKcs and the initiation of the DDR are triggered by their specific DNA binding co-factor complexes, which recruit and activate the respective kinase at the DNA lesion. Once activated, ATM, ATR and DNA-PKcs phosphorylate an overlapping pool of substrates to promote DNA repair and coordinate other DNA metabolism processes (transcription, replication, mitosis) [5, 10]. Among them, ATM and ATR are conserved in all eukaryotes, while DNA-PKcs has evolved only in vertebrates.

**Fig. 1:**
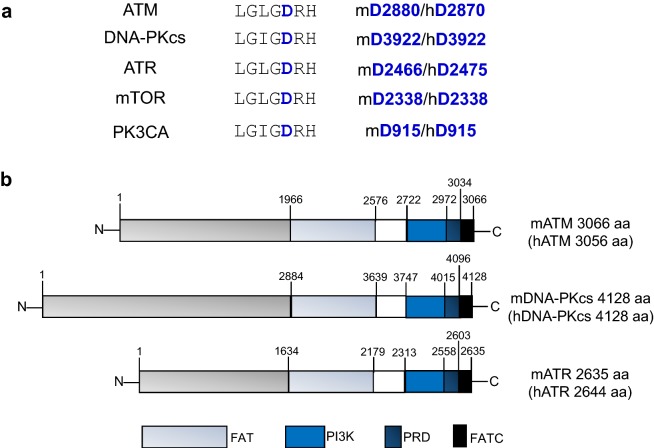
The domain structure and the conserved catalytic loop of PI3KKs. **a** The catalytic loop in the kinase domain, with the conserved aspartate (D) residue depicted in blue, is reported for ATM, DNA-PKcs, ATR, mTOR and PK3CA. The location of the D residue in the mouse (m) and human (h) proteins is also indicated. **b** Structural domain organizations of PI3KKs ATM, DNA-PKcs and ATR, with underlined amino acid positions. The number of amino acids in the mouse full length proteins, and corresponding human in brackets, is indicated

At the molecular level, ATM, DNA-PKcs and ATR are members of the PI3KKs family, which also includes mammalian target of rapamycin (mTOR) which regulates cellular metabolism, suppressor of morphogenesis in genitalia (SMG1) which functions in the nonsense mediated decay pathway, and the catalytically inactive transformation/transcription associated protein (TRAP) which regulates transcription [11]. All of the PI3KKs share some structural similarity within the PI3K kinase domain, especially the essential amino acids in the catalytic loop that coordinate ATP binding and stabilize the transition state of the phosphorylation reaction (Fig. 1a). The PI3KKs also have similar domain organization around the kinase domain, including the conserved FRAP-ATM-TRRAP (FAT) domain that precedes the kinase domain, the PIKK-regulatory domain (PRD) and the short FAT C-terminal motif (FATC) after the kinase domain [5] (Fig. 1b). The N-terminal region is made up by α-helical repeats of significantly variable size and shape [12] that interact with the DNA binding co-activator complex, namely MRE11-RAD50-NBS1 (MRN) for ATM kinase [13, 14], XRCC6/XRCC5 (hereafter KU70-KU86, KU80 in mouse, simply KU for the heterodimer) for DNA-PKcs [6, 15] and RPA-ATRIP for ATR [7, 16]. Each activator complex has a high affinity for different types of DNA lesions, explaining the specificity of activation of ATM, ATR and DNA-PKcs (discussed below). Once activated, the three kinases preferentially phosphorylate a serine or a threonine residue preceding a glutamine, referred to as the S/T-Q motif [17]. Given the small size of the motif, a large number of overlapping substrates have been identified through proteomic studies, which also include ATM, ATR and DNA-PKcs themselves (inter-molecular auto-phosphorylation and trans-phosphorylation). Mouse models with complete deletion of ATM, ATR and DNA-PKcs have been generated and elucidate the critical function of their kinase activity in embryonic and physiological development. In the past few years, we and others have generated a number of mouse models expressing kinase-dead (KD) ATM, ATR and DNA-PKcs [18–21]. In each case, the expression of KD-kinases causes much more severe genomic instability than loss of the kinases themselves. The phenotypes of these mouse models and the recently revealed high resolution structures [15, 16, 22–24] provide insights on the dynamic exchange of the kinases upon activation and the previously unappreciated link between catalysis and kinase exchange. The results revealed specific biological differences between kinase deletion and inhibition. In the following paragraphs, we will detail the activation pathway, and compare the unique features of mouse models with null or KD mutations of ATM, ATR and DNA-PKcs (Table 1) and attempt to summarize their common features and their implications in human disease and cancer therapy.

**Table 1:** Summary of currently available ATM, ATR and DNA-PKcs mouse models

Mouse models	Mutations	Fitness	Fertility	Main phenotypes	References
DNA-PKcs					
*DNA*-*PKcs*^−*/*−^	Null/knockout	Viable	Fertile	SCID; T and B cells developmental blockade; defective coding joint formation	Taccioli et al. [188], Gao et al. [49]; Kurimasa et al. [50]
*DNA*-*PKcs3A/3A*	Knockin, T2605/T2634/T2643A, phosphorylation site mutations	Viable	ND	Small; p53 dependent bone marrow failure; early lethality (2–3 weeks old)	Zhang et al. [74]
*DNA*-*PKcsKD/KD and*	Knockin, kinase dead DNA-PKcs (D3922A)	Embryonic lethal (E16.5)	ND	SJ and CJ fomation blocked; CSR defects; embryonic lethality rescued by KU deletion	Jiang et al. [20], Crowe et al. [62]
*DNA*-*PKcsKD/*−*					
*DNA*-*PKcsPQR/PQR*	Knockin, S2053 cluster mutated to Alanine	Viable	Fertile	Normal CSR and V(D)J recombination; moderate IR sensitivity	Jiang et al. [73]
*DNA*-*PKcsSD/SD*	Knockin, S2053 cluster mutated to Aspartate	Viable	Fertile	Normal CSR and V(D)J recombination	Jiang et al. [73]
ATM					
*Atm*−*/*−	Null/knockout	Viable	Infertile	Growth retardation; lack of mature gametes; T cells deficiency and thymic lymphomas	Barlow et al. [91], Elson et al. [92], Xu et al. [93], Borghesani et al. [94]
*AtmTgS1987A and AtmTgS1987A/S367A/S1899A*	BAC transgene, S1987A or S1987A/S367A/A1899A, phosphorylation site mutations	Viable	Fertile	No major phenotypes; proper DDR	Pellegrini et al. [99], Daniel et al. [100]
*AtmKD/KD and AtmKD/*−**	Knockin, kinase dead ATM (D2880A, N2885K)	Embryonic lethal (E9.5)	ND	Severe genomic instability; hyper sensitivity to Topoisomerase I inhibitors and pro-cancer	Yamamoto et al. [18, 121]
*AtmTgD2899A Atm*−*/*− *and AtmTgQ2740P Atm*−*/*−	BAC transgene, kinase dead ATM (D2899A, Q2740P)	Embryonic lethal (< E12.5)	ND	Severe genomic instability, PARP inhibitor sensitivity	Daniel et al. [19]
ATR					
*Atr*−*/*−	Null/knockout	Embryonic lethal (< E7.5)	ND	Chromosome fragmentation at blastocyst stage	Brown et al. [125]
					de Klein et al. [146],
*AtrSeckel/Seckel*	Seckel mutation (exons 8–10 replaced by human sequence with A ≥ G substitution in exon 9)	Viable	Fertile	Craniofacial abnormalities; growth retardation; embryonic replicative stress; accelerated aging	Murga et al. [152]
*Atr *+*/KD*	Knockin, kinase dead ATR (D2466A)	Viable	Male infertility	Male spermatogenesis defects, mild lymphocytopenia	Menolfi et al. [21]
*AtrKD/*−***	Knockin, kinase dead ATR (D2466A)	Embryonic lethal (< E9)	ND	Early embryonic lethality	Menolfi et al. [21]

*ND* not determined

* DNA-PKcs+/− and DNA-PKcs+/KD mice are viable and fertile

** Atm+/− and Atm+/KD mice are viable and fertile

*** Atr+/− mice are viable and fertile. AtrKD/KD mice cannot be obtained due to Atr+/KD male infertility

## The DNA damage response—DNA-PKcs, ATM and ATR

### DNA-dependent protein kinase catalytic subunit (DNA-PKcs)

DNA-dependent protein kinase (DNA-PK) was discovered as the gene mutated in mice with spontaneous T- and B- severe combined immunodeficiency (SCID) [25]. It was noted early on that the kinase activity of DNA-PK is stimulated by DNA, thus the name—DNA-dependent Protein Kinase [26, 27]. At the molecular level, DNA-PK holoenzyme includes the conserved DNA binding KU70–KU86 (KU80 in mouse) heterodimer (KU) and the vertebrate specific large catalytic subunit (DNA-PKcs). The crystal structure of full-length KU70 and KU80 without the flexible C-terminal domain shows that KU forms a ring, which allows dsDNA, regardless of terminal structure (*e.g.*, blunt, a hairpin or short overhangs) to thread through [28]. The stable double-ring structure formed by the KU heterodimer nicely explains its specificity to dsDNA ends. In the co-crystal structure with DNA, KU does not interact with any bases when fitting into the minor and major grooves formed by B-form DNA, which explains its sequence independent binding to DNA [28]. While the core of KU70–KU86 is conserved in all eukaryotes, the C-terminal globular domain of KU86 seems to co-evolve with DNA-PKcs in vertebrates. The very C-terminus of KU86 has conserved sequences that also exist in NBS1 (for ATM) and ATRIP (for ATR) and are required for DNA-PKcs recruitment and activation [29]. The exact mechanism by which DNA-binding of KU activates DNA-PKcs remains speculative even with the recently characterized Cryo-EM structure of the DNA-PK holoenzyme [22–24]. DNA-PKcs can undergo intermolecular auto-phosphorylation and also contribute to the residual IR induced phosphorylation of ATM substrates (*e.g.*, H2AX, SMC1 and KAP1) in ATM-deficient or inhibited cells [20, 30, 31], explaining their critical redundant function during embryonic development [32, 33] and DNA repair [34, 35].

In addition to this redundant role in DDR, KU and DNA-PKcs are members of the classical non-homologous end-joining (cNHEJ) pathway, one of the two major DSB repair pathways in mammalian cells [36]. It has been shown that the kinase activity of DNA-PKcs suppresses spontaneous and DSB-induced homologous recombination (HR), indeed channeling the lesions into the NHEJ pathway [37, 38]. The cNHEJ in vertebrates entails both end-processing (hairpin-opening) and end-ligation. Among the 9 cNHEJ factors identified thus far, KU70–KU86, DNA Ligase 4-XRCC4-XLF are conserved in all eukaryotes and are required for end-ligation. DNA-PKcs and Artemis evolved in vertebrates and the complete loss of DNA-PKcs or Artemis abolishes end-processing, especially hairpin opening, without completely abrogating end-ligation. The evolutionary origin of the newly identified cNHEJ factors PAXX and MRI remain elusive, but they both bind to the core region of KU and are required for end-ligation in XLF-deficient cells and mouse models [39–43]. During cNHEJ, KU binds to DNA [44], therefore stimulating the end-ligation by DNA ligase 4-XRCC4-XLF [45, 46]. DNA bounded KU also recruits DNA-PKcs, which in turn recruits and activates Artemis endonuclease for end-processing [47]. Activation of Artemis requires DNA-PKcs protein and the kinase activity from either DNA-PKcs or ATM in G1 arrested cells [20].

**Fig. 2:**
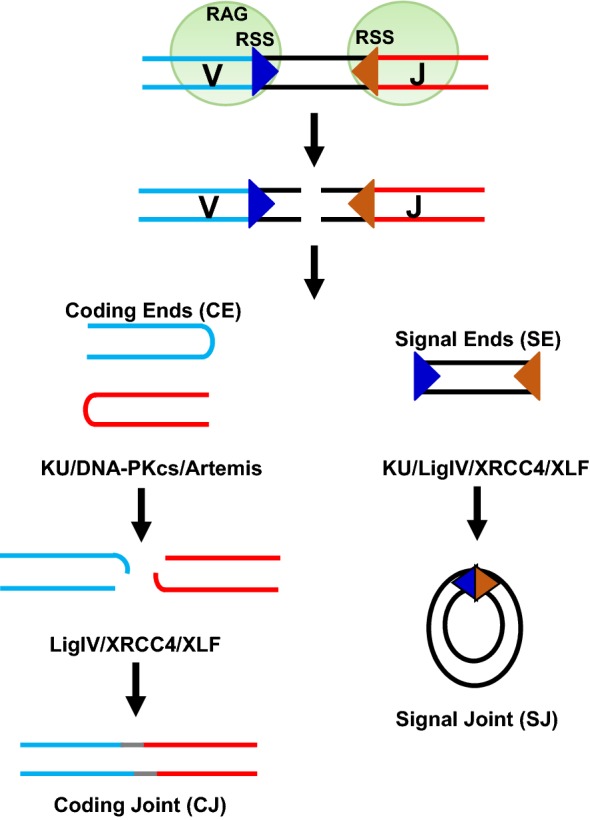
Schematic representation of V(D)J recombination. RAG endonucleases (RAG1 and RAG2) introduce DSBs between the participating V(D)J gene segments and their flanking recombination signal sequences (RSS). RAG cleavage generates a pair of blunt signal ends (SEs) and a pair of covalently sealed hairpined coding ends (CEs). While SEs are directly and precisely ligated by the cNHEJ factors Ligase IV/XRCC4/XLF to form a signal joint (SJ), the hairpin CEs have to first be opened by DNA-PKcs/Artemis and then joined through a process that can result in gain or loss of nucleotides and leads to the formation of a coding joint (CJ)

The end-processing and end-ligation phases of cNHEJ can be clearly distinguished in the context of V(D)J recombination, which assemble the functional antigen receptor genes from germline Variable, Diverse and Joining gene segments [48]. Lymphocyte-specific endonucleases—recombination activating genes, RAG1 and RAG2, initiate V(D)J recombination by introducing DSBs between participating V, D or J segments and their flanking recombination signal sequences (RSS) (Fig. 2). RAG cleavage generates a pair of hairpin coding ends (CEs) and a pair of blunt signal ends (SEs). While the two blunt and 5′ phosphorylated SEs can be directly joined via the end-ligation machinery of cNHEJ, the hairpin CEs have to be opened by DNA-PKcs and Artemis first, before ligation. Consistent with the critical role of DNA-PKcs in end-processing, DNA-PKcs null lymphocytes accumulate hairpin CEs, leading to T- and B- SCID in mouse models [49–51]. Meanwhile, DNA-PKcs null cells can ligate SEs to form signal joints (SJs) effectively with moderately reduced fidelity [20, 49], potentially reflecting an end-synapsis function of DNA-PKcs [52, 53]. Consistent with the non-essential role of DNA-PKcs in end-ligation, DNA-PKcs null mice are born of normal size with isolated immunodeficiency [49–51], in contrast to the embryonic lethality of end-ligation deficient *Xrcc4*^−*/*−^ or *Lig4*^−*/*−^ mice [54, 55]. DNA-PKcs deficient naïve B cells with pre-assembled immunoglobulin (Ig) genes can undergo fairly efficient class switch recombination (CSR)—a process to generate antibodies with different isotypes and thus effector functions [56–61]. Recent sequence analyses of the CSR junctions from DNA-PKcs null B cell reveal significant increases in micro-homology (MH), consistent with mild cNHEJ defects [62]. In this regard, all remaining CSR junctions of end-ligation defective B cells (*e.g.*, *Xrcc4*^−*/*−^ or *Lig4*^−*/*−^) are also enriched for MH [63, 64].

In sharp contrast to the normal development of DNA-PKcs null mice, a mouse model expressing kinase dead (KD) DNA-PKcs with a knockin D3922A mutation died in utero with extensive post-mitotic neuronal apoptosis [20], similar to *Lig4*^−*/*−^ or *Xrcc4*^−*/*−^ mice [54, 55, 65]. Indeed cNHEJ mediated end-ligation is completely abrogated in *DNA*-*PKcs*^*KD/KD*^ lymphocytes during V(D)J recombination and Ig CSR, resulting in severely defective and extensive resections and a significant enrichment of MH-mediated junctions [62]. Loss of KU rescued the embryonic lethality of *DNA*-*PKcs*^*KD/KD*^ mice and truncation of the KU80 C-terminal domain partially restored end-ligation [20], suggesting that once recruited to the DNA ends, DNA-PKcs physically blocks end-ligation in the absence of its kinase activity. This unexpected end-protection role of DNA-PKcs is also supported by the ability of purified DNA-PK holoenzyme, but not KU, to block DNA end-ligation by T4 DNA-ligase in the absence of ATP [66]. The lack of detectable end-ligation defects in *DNA*-*PKcs*^+*/KD*^ cells and mice is potentially consistent with the intermolecular auto-phosphorylation of DNA-PKcs at each ends of DSBs.

DNA-PKcs is the best characterized substrate of itself [67, 68]. Two phosphorylation clusters (S2023-S2056 and T2609-T2647) precede the FAT domain and an auto-phosphorylation site (T3950) within the kinase domain have been characterized [69]. Upon radiation, the S2056 cluster is primarily phosphorylated by DNA-PKcs itself [70]. Following IR or UV, T2609 is phosphorylated by ATM and ATR, respectively [71, 72]. Impaired phosphorylation at either or both clusters increases IR-sensitivity in CHO cells with ectopic expression of DNA-PKcs [70]. Yet, alanine substitution at the S2056 cluster (corresponding to S2053 in mouse) does not affect V(D)J recombination or CSR, and only causes moderate IR sensitivity in B cells [73]. Meanwhile, alanine substitution at the T2609 cluster (mouse T2605A/T2634A/T2643A, *DNA*-*PKcs*^*3A*^) caused lethal p53-dependent bone marrow failure [74] without abolishing chromosomal V(D)J recombination [75]. Moreover, the residual end-ligation in both *DNA*-*PKcs*^*3A/3A*^ and *DNA*-*PKcs*^−*/*−^ cells requires ATM kinase activity, similar to that in *Xlf*^−*/*−^ cells, implying the DNA-PKcs and its phosphorylation at T2609 might contribute to synapsis. Nevertheless, the severe end-ligation defects in *DNA*-*PKcs*^*KD/KD*^ cells and the much more moderate, if any, end-ligation defects in mice expressing phosphorylation-defective DNA-PKcs suggest that the catalysis itself might regulate the end-protection role of DNA-PKcs beyond phosphorylation. Similarly, the phosphorylation site mutations of ATM also yield different results than the kinase dead mutations, suggesting the catalysis, not necessarily the auto-phosporylation, might regulate the conformation changes of the kinases.

Finally, despite the normal development of mice or horses in the absence of DNA-PKcs or KU, cultured human cells, including cancer cells, cannot tolerate the loss of DNA-PKcs or KU [76, 77]. This essential function of DNA-PKcs and KU in human cells seems to be independent of cNHEJ, since (1) the protein levels of KU and DNA-PKcs increase 50 fold in human cells independently from all the other NHEJ factors [73], (2) the loss of LIG4 or XRCC4 can be well tolerated in cultured human cells [78]. Correspondingly, DNA-PKcs protein expression is preserved in the two patients with DNA-PKcs deficiency identified thus far—one patient carries the L3062R mutation in the FAT domain, with preserved kinase activity and isolated SCID [79], and the other one has reduced kinase activity with SCID and severe microcephaly [80], similar to patients with hypomorphic mutations in LIG4 or XRCC4 [81–83]. Telomere instability has been implicated [76, 77] and purified yeast KU binds to the RNA template of telomerase [84, 85]. While characterizing the spontaneous tumors in the *DNA*-*PKcs*^*KD/KD*^ *Tp53*^−*/*−^ mice, we and others uncovered an unexpected role of DNA-PKcs in hematopoiesis [187]. While loss of DNA-PKcs or KU did not cause immediate bone marrow failture, expression of kinase-dead or phosphorylation defective (T2609A) DNA-PKcs compromised ribosomal RNA processing, protein translocation and hematopoiesis, and mice models with alanine substitution at all five (*DNA*-*PKcs*^*5A/5A*^) or three (*DNA*-*PKcs*^*3A/3A*^) out of the five threonine residues at the T2609 cluster succumbed to lethal anemia by 4 weeks [74, 187]. Strikingly, loss of KU fully rescued the anemia in *DNA*-*PKcs*^*5A/5A*^ mice, suggesting a cNHEJ independent function of DNA-PKcs in erythrocyte differentiation and protein translation. In this context, we and others found that KU as well as DNA-PKcs gather in nucleoli in a detergent resistant manner independent of other cNHEJ factors [86, 187]. Using UV crosslink, a large number of KU and DNA-PKcs interacting physiological RNAs have been identified, including the rRNA itself and the small nucleoli RNA (snoRNA) U3, that has been implicated in rRNA processing [187]. In silico folding analyses suggested that KU and DNA-PKcs bind to a stem-loop of U3. In vitro, this U3 stem-loop can activate DNA-PKcs and trigger T2609 phosphorylation. Thus, this study uncovered a cNHEJ independent role of DNA-PKcs on structured RNA [187]. Notably, the telomerase RNA template is also partially processed in the nucleoli. While whether this RNA-dependent function of DNA-PKcs explains the need of DNA-PKcs in human cells remains to be examined, these findings open up a new function for DNA-PKcs beyond cNHEJ.

### Ataxia-telangiectasia mutated (ATM)

ATM stands for ataxia-telangiectasia mutated. Homozygous germline inactivation of ATM causes the Ataxia-telangiectasia (A-T) syndrome, characterized by oculocutaneous telangiectasia (vascular dilation), cerebellar ataxia, immunodeficiency, greatly increased risk for cancer, especially lymphoid malignancies, and extreme sensitivity to ionizing radiation (IR) [87–89]. The vast majority (~ 90%) of A-T patients carry compounded truncating or frameshifting mutations with low or little ATM protein expression. The low frequency of the missense mutations is surprising given the large size of ATM (3056aa) and the well-recognized importance of the kinase domain at the C-terminal. The nearly 1% carrier rate in some populations and the occurrence of A-T in 1 out of 40,000 to 100,000 live births [90] suggest potential embryonic lethality or underdiagnoses. This apparent discrepancy was solved when two mouse models expressing kinase-dead ATM were found to be embryonic lethal [18, 19], while four independently generated *Atm* null mice were born at the expected ratio and recapitulated many features, including the immunodeficiency and lymphoma phenotypes, of A-T patients [91–94].

At the molecular level, ATM is activated by the MRN complex at the site of DNA damage. Unlike KU, which activates DNA-PKcs with as short as 30nt of DNA, MRN mediated activation of ATM is optimal with DNA longer than 2 Kb [8]. Recent single molecule experiments show that the MRN complex slides along the dsDNA and activates ATM when MRN hits a road block, such as KU at the DNA ends [95]. Prior biochemical studies also suggest that MRN unwinds the DNA ends and activates ATM via the ssDNA region [14]. Like in the case of KU-DNA-PKcs, the C-terminal domain of NBS1 contains a unique motif, which forms one of the direct contact points with ATM [13] during activation. Activation of ATM has been linked to a dimer/oligomer to monomer transition and intermolecular auto-phosphorylation (human S367, S1893, S1981, S2996 and potentially other sites) [14, 96, 97]. In human cells, loss of one or several autophosphorylation sites compromises ATM activation [96, 98]. Yet, mouse models with alanine substitution at S1987 or at two other additional auto-phosphorylation sites are virtually normal [99, 100], leading to a dilemma regarding the function of the auto-phosphorylation sites. Nevertheless, S1981 phosphorylation has been a valuable and widely used marker for ATM activation in cells. As a master regulator of DSB-induced DDR, ATM plays a critical role in DNA repair in part by phosphorylating the C-terminal tail of the histone variant H2AX on Serine 139, which serves as a hub to recruit MDC1 and eventually initiate the ubiquitination cascades [101]. In addition, ATM also orchestrates cell cycle checkpoints by phosphorylating CHK2 kinase [102] at the G2/M transition and p53 Serine 15 (Serine 18 in mouse) at the G1/S transition [103, 104]. The checkpoint function of ATM plays an important role in tumor suppression, evidenced by the strong mutual exclusivity of ATM and p53 mutations in human cancers.

Mouse models with complete loss of ATM protein, generally referred to as *Atm*^−*/*−^ mice hereafter [91–94], were born at the expected mendelian ratio and viable. They recapitulate many clinical features of the A-T patients, including growth retardation (~ 25% smaller), male and female infertility due to meiosis failure [105], and moderate yet consistent immunodeficiency in both T cell development [94] and B cell Immunoglobulin CSR [106, 107]. A-T patients have increased risk for lymphoid malignancies. In some genetic backgrounds, by 4 months of age, nearly all *Atm*^−*/*−^ mice succumbed to T cell malignancies, with recurrent t(12;14) translocations that are syntenic to the chromosome 14 inversions found in the peripheral T cells of A-T patients [108, 109], suggesting a role of ATM in the repair phase of chromosomal V(D)J recombination. Correspondingly, loss of RAG endonuclease that initiates V(D)J recombination, delays thymic lymphoma in *Atm*^−*/*−^ mice [110, 111]. Using a chromosomal V(D)J recombination reporter, it was identified a role of ATM in stabilizing the post-cleavage complex [112], which is further exacerbated by the loss of XLF, a NHEJ factor [113]. Although neurons from *Atm*^−*/*−^ mice are sensitive to IR, *Atm*^−*/*−^ mice do not develop spontaneous Ataxia as A-T patients [94, 114–116]. Despite normal development, cultured *Atm*^−*/*−^ murine fibroblasts enter p53-dependent senescence within 5 passages [91]. Low oxygen cultures significantly delay the senescence of *Atm*^−*/*−^ mouse embryonic fibroblasts (MEFs), suggesting ATM might have a role in anti-oxidative stress responses. In this context, heterozygous *Atm*^+*/*−^ mice also show increased sensitivity to IR, accompanied by premature ageing and decreased survival [117]. Although the physiological targets and regulators of ATM during oxidative stress remain elusive, purified ATM can be activated by reactive oxygen species (ROS) in a process that requires disulfate bond formation between C2991 residues of two ATM monomers [118].

In 2012, three mouse models expressing catalytically inactive ATM protein at similar levels to endogenous ATM were made—the transgenic D2899A or Q2740P, and the knockin D2880A [18, 19]. The kinase-dead ATM, when expressed solely in the absence of wildtype ATM, causes severe genomic instability and embryonic lethality at E9.5–10.5. In comparison to the null allele, expression of ATM-KD does not further compromise lymphocyte development or lymphocyte specific gene rearrangements, but instead increases chromatid breaks that are consistent with replicative or post-replicative DNA damage. Accordingly, in comparison to *Atm*-null cells, *Atm*^*KD/*−^ cells are more sensitive to genotoxic agents that selectively induce replication associated breaks, such as topo-isomerase I inhibitors and cross-linking agents, but are similarly sensitive to classical DSB generating IR or topoisomerase II inhibitors. Moreover, *Atm*^*KD/*−^ cells, but not *Atm*-null cells, display defects in homologous recombination using a DR-GFP reporter assay [119, 120]. Although the exact DNA damage structure that is sensitive to the expression of ATM-KD remains unknown, the phenotypes of the *Atm*^*KD/*−^ cells suggest that it is likely replication related, and not simply DSBs dependent. Heterozygous *Atm*^+*/KD*^ mice are fertile, of normal size and somatic deletion of the conditional allele in *Atm*^*C/KD*^ mice causes earlier and more aggressive lymphomas [121]. This might explain the increased cancer risk of ATM mutation carriers [122]. The embryonic lethality of the *Atm*-*KD* models might also explain the lack of missense ATM mutations in germline A-T patients. Indeed, over 70% of somatic ATM mutations identified in TCGA databases are missense mutations. Although the functional impact of most ATM missense mutations remains unclear, the more than threefold enrichment in the kinase domain suggests that at least a subset of them would compromise ATM kinase activity [121]. The embryonic lethality of the *Atm*-*KD* mice also brought up the question about the nature and the impact of the ~ 10% missense mutations of ATM in A-T patients. Among them, a subset might retain some minimal ATM kinase activity or have low expression of ATM. One of the former examples is the inframe deletion 7636del9 [123]. The mouse model with the corresponding mutation is also viable, expresses Atm with minimal activity, and lives long enough to develop B cell lymphomas and sarcomas beyond the thymic lymphomas [124]. Alternatively, a subset of the missense mutations might express kinase dead ATM that cannot interact with MRN, thus not eliciting the toxic structural functions.

### Ataxia-telangiectasia and Rad3 related (ATR)

Ataxia-telangectasia and Rad3 related (ATR) kinase forms obligatory tetramers with two ATR and two ATR interacting proteins (ATRIP). ATRIP is essential for ATR protein stability. ATR kinase is dispensable for G0/G1 arrested cells, but essential for proliferating cells, especially during normal replication [125, 126]. ATR activation requires ssDNA coated by the heterotrimeric complex Replication Protein A (RPA) [7]. In addition to resected DSBs, R-loops generated during transcription, DNA replication stresses induced by polymerase poisons and deoxyribonucleotide triphosphate (dNTPs) depletion can all activate ATR [127]. ATRIP directly interacts with RPA on ssDNA to recruit and activate ATR [7]. Unlike ATM and DNA-PKcs, full ATR activation requires additional factors, like RAD17, RAD9-RAD1-HUS1 (9-1-1 complex) [128] and topoisomerase II binding protein 1 (TOPBP1) [129] or RPA and Ewing tumor-associated antigen 1 (ETAA1) [130–133]. Specifically, TOPBP1 and ETAA1 contain an ATR-activating domain (AAD) and serve as the allosteric activators of ATR [134]. Recent evidences suggest that TOPBP1 is required for ATR activation during replication stresses, while ETAA1 is more important for ATR dependent S/G2 checkpoint activation during unperturbed cell cycle progression [135, 136]. Activated ATR phosphorylates and activates its downstream kinase CHK1 [137, 138]. CHK1 is a relatively specific substrate of ATR upon replication stress, but can also be phosphorylated by ATM or DNA-PKcs in the absence of ATR kinase activity [21, 139]. The ATR-CHK1 axis activates the G2/M cell cycle checkpoint through phosphorylation and inactivation of CDC25 phosphatases, prevents unscheduled origin firing, maintains replication fork stability and regulates nucleotide availability, thereby playing critical roles in coordinating replication fork progression, cell cycle and DNA repair [127]. Correspondingly, ATR is important for the stability of regions that are difficult to replicate, including fragile sites [140], repetitive regions (*e.g.*, microsatellites and quasi-palyndromic AT-rich repeats) [141] and telomeres [21, 142]. ATR was also found to be activated by R-loops forming at centromeric regions during mitosis, uncovering a role of ATR beyond S phase [143].

ATR, and its effector kinase CHK1, are essential for proliferating cells. At the cellular level*, Atr*^−*/*−^ blastocysts display severe chromosome fragmentation consistent with mitotic catastrophe, suggesting that the role of ATR in preventing premature mitotic entry is critical for early embryonic development [125]. Concomitant loss of *TP53* exacerbates the mitotic catastrophe associated with the loss of ATR or CHK1 [144], likely due to the important role of p53 in initiating the late G2/M checkpoint [145]. This has been the genetic basis to use ATR or CHK1 inhibitors to preferentially target p53 deficient cancer cells. Accordingly, complete loss of ATR and CHK1 leads to E7.5-E8.5 embryonic lethality in mice [125, 146–148]. An inactivating point mutation in the AAD of TopBP1 (W1147R), required for full activation of ATR kinase, also results in embryonic lethality [149]. Hypomorphic mutations of ATR, like the splicing mutation A2101G that leads to extremely low levels of the protein, underlie a subset of Seckel syndrome patients with craniofacial abnormalities, microcephaly, and growth retardation [150, 151]. Several mouse models were made to mimic the ATR-associated Seckel Syndrome. Among them, the one carrying humanized exons 6–8 with the A2101G splicing site mutation (*Atr*^*s/s*^) [152] recapitulated many features of the corresponding human patients, including low birth rate, craniofacial abnormalities, growth retardation and microcephaly, and lethal progeroid that was aggravated by *TP53* deletion. Increasing dNTP levels through increased expression of the catalytic subunit of RNR (Ribonucleotide Reductase) Rrm2, which converts NTPs in dNTPs, partially rescues the accelerated aging and replication defect phenotype associated with Seckel mice and cells, suggesting nucleotide concentration might contribute to replication stress in general or in the absence of ATR especially [153].

Somatic inactivation of ATR via a conditional allele and a tissue specific Cre revealed an essential role of ATR in stem cell maintenance and tissue homeostasis, underlying a number of premature aging phenotypes [154]. Moreover, double mutant *Atr TP53* cells display high levels of chromosome fragmentation and mitotic catastrophe, not compatible with viability [155]. Based on this synergistically lethal phenotype, ATR (and CHK1) inhibitors have been used in the treatment of p53-deficient human cancers. In mouse models, 90% reduction of ATR expression level is well tolerated and does not affect normal tissue homeostasis, but inhibits the growth of different cancers overexpressing Ras or c-Myc, regardless of p53 status [156].

A mouse model expressing kinase-dead ATR D2466A (D2475 in human), *Atr*^+*/KD*^, develops normally [21]. Nevertheless male *Atr*^+*/KD*^ mice, but not female *Atr*^+*/KD*^, are sterile, preventing the generation of double mutant mice *Atr*^*KD/KD*^. This is in contrast to the normal fertility of both male and female *Atr*^+*/*−^ mice [126]. The presence of ATR-KD protein prevents efficient phosphorylation of H2AX at X–Y bodies during meiotic pachytene, a process necessary for the transcriptional silencing of X–Y chromosomal associated genes required for spermatogenesis [157, 158]. Analyses of ATR Seckel mice or mice pharmacologically treated with ATR inhibitors also identified a role of ATR in X–Y meiosis recombination [159, 160]. Mechanistically, ATR-KD protein displays a dominant negative function in limiting the dynamic exchange of ATR itself and a subset of RPA bound to ssDNA [21]. As a consequence, ATR-KD expressing cells display genomic instability at regions with accumulation of single-stranded DNA, like X–Y bodies, telomeres and ribosomal DNA, consistent with the essential role of ATR in replication, but not at DSBs. Differently from ATM- or DNA-PKcs deficient mice, *Atr*^+*/KD*^ mice display only a mild lymphocytopenia and subtle CSR defects, likely due to an effect on cell cycle and proliferation and not on NHEJ, which is independent of ATR, since it requires minimal, if any, ssDNA generation. And while ATM-KD and DNA-PKcs-KD do not show any clear dominant negative effect, ATR-KD does, probably due to the stable hetero-tetramer formed by two molecules of ATR and two molecules of ATRIP, where a single ATR-KD protein might directly interfere with the normal activity of the other WT ATR subunit, inhibiting its exchange on DNA, inter-molecular autophosphorylation and the canonical downstream functions (*i.e.*, CHK1 phosphorylation).

### PI3KKs inhibition, synthetic lethality and cancer therapy

Genomic instability and defects in the DNA Damage Response pathways have long been connected to cancer etiology and pathogenesis [161]. Several genes involved in DNA damage induced cell cycle arrest (*p53, Atm, Chk1, Chk2*) are main tumor suppressor genes. Partial or complete inactivation of these genes allows cell to tolerate on-going genomic instabilities and to proliferate in the presence of DNA damage. On the other hand, transformed cancer cells also rely more on DDR proteins to cope with oncogene induced replication stresses. These findings led to the development of specific inhibitors against DNA damage response kinases to target the vulnerability of cancer cells and sensitize them to genotoxic cancer therapy [162].

ATM is a tumor suppressor gene that is inactivated in 2–8% of common epithelial cancers, including breast and pancreatic [122, 163]. ATM is also biallelically lost in nearly all T cell prolymphocytic leukemias (T-PLL), 50% of mantle cell lymphomas (MCL) and 5–10% of chronic lymphocytic leukemias (CLL) [164–167]. Loss of ATM renders cancer cells sensitive to multiple genotoxic therapies, including traditional chemotherapy, as well as the newly developed PARP inhibitors [168]. Given the redundant role of ATM, ATR and DNA-PKcs in the DNA damage response, ATM null tumors are also hypersensitive to DNA-PKcs or ATR inhibition, in agreement with the synergistic lethality of *Atm*^−*/*−^ *Prkdc*^−*/*−^ double knockout mice [32, 33]. DNA-PKcs inhibition has been effective as a monotherapy in ATM-deficient B cell lymphomas in cell lines and pre-clinical models [169]. Mutations in *MSH3*, a mismatch repair (MMR) gene frequently mutated in colorectal and endometrial cancer, were found to be hypersensitive to DNA-PK and PI3K dual specific inhibition—KU-0060648 and siRNA mediated deletion of DNA-PKcs or KU [170]. HR and MMR are inextricably linked and MMR compoments play an important role in ensuring faithful homology is used during HR. The hypersensitivity of the *MSH3*-deficient cancer to DNA-PK inhibition might be exploited in cancer therapy.

Complete loss of DNA-PKcs or ATR are rare in cancers. Rather, ATR activity becomes critical for the survival of Myc or Cyclin E deregulated cancers with oncogene-induced replication stress [171, 172]. Specific ATR inhibitors (ATRi), such as VE-821 and AZD6738, sensitize diverse cancer cell models to genotoxic agents, including cross-linking agents, topoisomerase I or II inhibitors, PARP inhibitors and the nucleoside analog gemcitabine [173–177], by allowing premature mitosis with incomplete DNA replication or chromosome separation. For similar reasons, cancer cells deficient for other G2/M checkpoint regulators—ATM or TP53, are also hypersensitive to ATR inhibition [178–180]. Furthermore, defects in other repair factors that would increase replication associated DNA damage, like loss of XRCC1 or ERCC1, also make cells more sensitive to ATRi in cell line models [181, 182]. Moreover, even PARPi-resistant BRCA1-deficient cancer cells are sensitive to ATR inhibition [183], in part due to the role of ATR in supporting BRCA1-independent loading of RAD51. Altogether, these observations suggest ATR inhibition might be synergistically lethal to cancer cells with various replication vulnerabilities.

## Conclusions

In addition to blocking the catalytic activity of their respective kinases, the use of kinase inhibitors lead to the observations that the inhibition might impact genomic instability beyond the loss of the kinases themselves. In this context, the mouse models expressing kinase-dead ATM, ATR and DNA-PKcs have provided strong genetic evidence for a structural function of these kinases at the DNA damage sites. In general, mouse models expressing kinase-dead PI3KKs present more severe genomic instabilities and development defects than the corresponding knockout mice. While loss of ATM and DNA-PKcs is compatible with life, expression of ATM-KD and DNA-PKcs-KD proteins leads to early embryonic lethality. Deletion of their sensor proteins, MRN for ATM and KU for DNA-PKcs, partially relieves the additional DNA repair defects, consistent with a model in which the catalytically inactive kinase physically blocks DNA repair at the site of DNA damage. Similarly, the expression of the ATR-KD protein displays selective toxicity during processes with accumulation of RPA-coated ssDNA. While some of the studies were initially motivated by the well-characterized auto-phosphorylation sites, the alanine substitution on ATM or DNA-PKcs have either yielded no phenotypes or very different phenotypes [74, 99, 100], suggesting loss of auto-phosphorylation is likely mechanistically different from kinase inhibition.

**Fig. 3:**
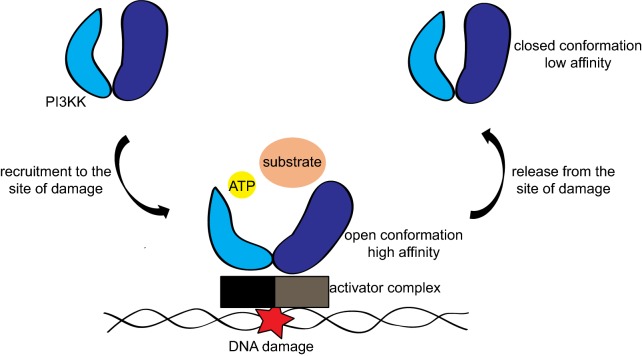
Proposed mechanism for the activation and deactivation cycle of PI3KKs. PI3KKs are recruited to the site of damage by their respective activator complexes and they assume an open conformation that allows ATP catalysis and substrate phosphorylation. Once deactivated, the kinases are recycled, losing their affinity for the activator complexes, thereby physically leaving the site of damage in a closed conformation. Kinase-dead proteins are likely stuck on the DNA, impeding the proper repair of the DNA lesion

Understanding the mechanism of kinase activation and exchange would be of valuable importance for comprehending the therapeutic effects and toxicities of the DDR inhibitors that are entering the clinics. Several pieces of evidence have suggested that PI3KKs dynamically exchange at the site of damage and that the kinase activity is important for this process [21, 68]. One possibility is that the conformational changes associated with kinase activation directly regulate the recruitment and exchange of these kinases at the DNA damage sites. Future studies are required to mechanistically uncover how the kinase activity and structural changes associated with its activation influence the recruitment to the DNA damage sites as well as the recycling and deactivation processes. Structural analyses suggest that the activation of the kinase and the engagement with substrate and ATP might provoke a higher affinity of the kinase to the activation complexes and DNA lesions. Once the catalytic reaction is completed, the kinase might return to a closed and lower affinity conformation to allow the protein to be recycled and physically leave the site of damage (Fig. 3). The presence of the ATP mimetic kinase inhibitors or the presence of the D-to-A kinase dead mutations potentially trap the kinase in complex with ATP and its substrate in a high affinity mode, where it blocks DNA repair and other DNA metabolism events. This model is consistent with the recent structural studies for ATR [16] and DNA-PK [15, 22–24], which depict a well-oriented catalytic center and one or two alpha-helices that guard the access to the catalytic center while interacting with the activation complex. A similar conformation has been noted for mTOR earlier on [184]. Recent structural analyses of PARP1, which is also activated upon interacting with DNA lesions, show remarkable similarity [185, 186], where a partial unfolding of the helix domain serves as the gate to the catalytic center. Genetic models and biochemical analyses of mutations affecting the gate-keeper domains are needed to further prove such a model. Understanding the allosteric changes associated with kinase activation would provide further insights on the design and use of specific kinase inhibitors. To date, the complete spectrum of the interplay between ATM, DNA-PKcs and ATR, has not been fully uncovered, but could provide a better understanding of the synthetic lethality and of the synergistic effects of ATMi, DNA-PKcsi and ATRi in cancer therapy.

## Data Availability

Not applicable.
